# Aluminium Drinking Water Treatment Residuals and Their Toxic Impact on Human Health

**DOI:** 10.3390/molecules25030641

**Published:** 2020-02-02

**Authors:** Izabela Krupińska

**Affiliations:** Faculty of Civil Engineering, Architecture and Environmental Engineering, Institute of Environmental Engineering, University of Zielona Góra, 15 Prof. Z. Szafrana St, 65-516 Zielona Góra, Poland; i.krupinska@iis.uz.zgora.pl; Tel.: +48-68-3282560

**Keywords:** water treatment plants, residual aluminium, coagulation, aluminium sulphate (VI), sodium aluminate, polyaluminium chlorides, monomeric Al species (Al_a_), medium polymerised Al species (Al_b_), colloidal Al species (Al_c_)

## Abstract

Aluminium exerts undeniable human health effects, so its concentration should be controlled in water treatment plants. The article presents and discusses the results of studies on the influence of selected properties of aluminium coagulants on the concentration of aluminium remaining in the purified water. The coagulants used were classical hydrolysing aluminium salts: aluminium sulphate (VI) and sodium aluminate as well as pre-hydrolysed polyaluminium chlorides: Flokor 105B and PAX XL10 that had different the alkalinity coefficient r = [OH^−^]/[Al^3+^]. The Al species distribution in the coagulants samples were analysed by the Ferron complexation timed spectrophotometry. On the basis of their reaction rates with ferron reagent, the aluminium species were divided into three categories: monomeric (Al_a_), medium polymerised (Al_b_) and colloidal (Al_c_). The usefulness of the tested aluminium coagulants due to the concentration of residual aluminium and dissolved aluminium, which is easily assimilated by the human body, was increased according to the following series: sodium aluminate (Al_a_ = 100%, Al_b_ = 0) < aluminium sulphate (VI) (Al_a_ = 91%, Al_b_ = 9%) < PAX XL 10 (Al_a_ = 6%, Al_b_ = 28%, r = 2.10) < Flokor 105B (Al_a_ = 3%, Al_b_ = 54%, r = 2.55).

## 1. Introduction

Aluminium is found in all natural waters and waterworks. It may occur in the form of organic and inorganic compounds in dissolved and undissolved form, and an important factor that affects its form of occurrence is the pH value [1,2]. It is amphoteric, combining with both acid and bases to form, respectively, aluminium salts and aluminates. The chemical nature of aluminium in water is essentially the chemistry of Al(OH)_3_ that has an amphoteric character and tendency to form complex ions and polymerise. Evidence has been provided by chemical modelling that in solutions with a pH below 5 aluminium exists predominantly as Al(H_2_O)_6_^3+^, with rising pH an insoluble Al(OH)_3_ complex forms at circumneutral pH that re-dissolves at higher pH as the Al(OH)_4_^−^ (aluminate) complex. The amount of aluminium in natural waters varies from 0.0001 to 1 mg/dm^3^, and in acidic waters (pH < 5) the concentration of aluminium may even exceed 100 mg/dm^3^. Aluminium compounds show low solubility in the pH range of 6–8, therefore in surface and subsurface waters the concentrations of aluminium are very low and are classified in the range of 60 to 300 µg/dm^3^ [3]. Abnormal aluminium concentrations in tap water may be caused by improper coagulation with aluminium coagulants. It is important to ensure that the amount of aluminium remaining in water intended for human consumption is as low as possible, given that elevated concentrations of aluminium may pose a potential risk to human health, resulting in brain changes characteristic of Alzheimer’s disease. Thus, the use of aluminium salts as a flocculants to purify drinking water has long been criticised [3]. The relationship between aluminium exposure and Alzheimer’s disease has been the focus of intense research, and epidemiological investigations have found a strong correlation between the accumulation of aluminium in the brain and Alzheimer’s disease, both for workers occupationally exposed to aluminium and for people who drink tap water that might contain higher aluminium content after being purified with aluminium salts [4,5]. With regard to drinking water exposure, an important question is whether the aluminium is derived from natural sources for instance from ingestion of clay minerals or as a consequence of water treatment methods. Water treatment using aluminium sulphate (VI) generally increases the percentage of dissolved, low molecular weight, polyaluminium species that are chemically reactive and possibly more readily absorbed [4]. Aluminium has been found in the brain of Alzheimer’s patients in the amyloid deposits characteristic of this disease. Aluminium may enter the brain through multiple routes: from blood, either through choroid plexus or across the blood brain barrier and from the nasal cavity into olfactory nerves, followed by direct distribution into the brain. The exact mechanism of aluminium toxicity on brain cells is not known, but there are several lines of evidence showing that this simple trivalent cation, incapable of redox changes, might exacerbate oxidative events and activate reactive oxygen species generation, linking aluminium storage to the pathogenesis of Alzheimer’s disease. Aluminium can also accumulate in the bones, causing their excessive brittleness and softening, resulting in osteomalacia with fracturing osteodystrophy [4,5,6]. Aluminium aqua-complexes can be ranked according to increasing toxicity in the following series [7]: [Al(OH)_2_^+^ ] < [Al(OH)^2+^] < [Al(H_2_O)_6_]^3+^. Already in the 1980s it was found that aluminium ions from water can diffuse into the blood plasma causing damage to the nervous system, and the penetration of ions through the blood–brain barrier depends on the degree of ionisation of the compound, lipid solubility and molecular size. Subsequent studies have shown that the dynamics of aluminium penetration into the brain and kidneys are of great importance. Atrophic lateral sclerosis, Parkinsonism associated with dementia are the next diseases whose cause is largely considered to be aluminium. The common feature of these diseases is atrophy of neurons, neurofibril degeneration, lymphopenia and T lymphocyte functioning disorders [5,6]. Aluminium is dangerous for living organisms, especially in acidic, but also alkaline environment because it occurs in ionised form and is then easily absorbed. In an inert environment, aluminium compounds are insoluble and excreted from the body [8,9]. The Environmental Protection Agency (EPA) in 2017 has recommended a Secondary Maximum Contaminant Level (SMCL) of 0.05–0.2 mg/dm^3^ for aluminium in drinking water [10]. World health organization (WHO) demanded the residual Al concentration in drinking water must be lower than 0.2 mg/dm^3^ [11], and in some countries, the rules are even stricter. The suggested adverse effect of aluminium on human health and its acceptable concentration in water intended for human consumption in Poland ≤ 0.2 mgAl/dm^3^ [12] in France, Canada, Japan and Sweden ≤ 0.1 mgAl/dm^3^ [1] and in the United States ≤ 0.05 mg/dm^3^ [13] in oblige to ensure a minimum content of aluminium in water intended for human consumption. A lot of works [1,2,7,8,9] advocate a tolerable threshold limited to 0.1 mg/dm^3^ in drinking water, while remaining vigilant about monitoring the waters treatment plants. The inorganic monomeric aluminium represents their main fraction of aluminium after water treatment (62% of total). The analysis of literature information [13,14,15,16,17,18,19,20] indicates that pre-hydrolysed aluminium coagulants are more useful in the treatment of water intended for human consumption than non-prehydrolysed ones such as aluminium sulphate (VI) or sodium aluminate. Pre-hydrolysed aluminium coagulants can be formed in a variety of ways, but there are two basic methods of manufacture. One starts with AlCl_3_ solution and adds a base such as caustic or lime to partially neutralise AlCl_3_. Total neutralisation will form Al(OH)_3_, but partial neutralisation will form polymeric aluminium compounds. The second method starts with Al(OH)_3_ or aluminium trihydrate and adds acid to form a partially neutralised aluminium compound. The methods produce different aluminium species that affects the overall coagulation properties. However, there is no clear information on the role of the degree of preliminary hydrolysis of these reagents in their usefulness for water purification due to the concentration of aluminium remaining in the water. The most frequently used coagulant in water treatment plants is aluminium sulphate (VI). Aluminium sulphate (VI) and sodium aluminate, when added to water, first undergo dissociation and then their cations hydrolysis. Aluminium sulphate (VI) after introduction into water and dissolution undergoes acid hydrolysis and only in a small range of pH from 6.8 to 7.2 it forms flocs. If the pH value is lower than 6.8 or higher than 7.2, aluminium sulphate (VI) does not coagulate completely. Sodium aluminate that undergoes alkaline hydrolysis does not cause acidification of the treated water as aluminium sulphate (VI). The type of hydrolysis product of aluminium sulphate (VI) and sodium aluminate depends on the pH and time of hydrolysis and therefore monomers may be formed: Al^3+^, Al(OH)_2_^+^, Al(OH)^2+^, Al(OH)_3_ and Al(OH)_4_^−^ as well as the polymers with the general formula Me_n_(OH)_y_^3n−y^ [20,21,22,23] determine the quantitative share of individual hydrolysis products outside the hydrolysis conditions, to a large extent depending on the type of coagulant. Hydrolysis of aluminium cations, which are products of dissociation of non-prehydrolysed coagulants such as aluminium sulphate (VI) or sodium aluminate, takes place immediately after their contact with water. As a result, the precipitation of the final hydrolysis products is faster than the reaction of the intermediate hydrolysis products with the colloids present in the treated water. When using non-prehydrolysed primary aluminium coagulants such as e.g., aluminium sulphate (VI), the formation of polycationic aluminium forms could prolong the hydrolysis time, which is only possible by acidification of the water before coagulation to pH < 6.0 [19]. However, such a solution complicates the technological system of water treatment and intensifies its corrosive aggressiveness. The pH is one of the main factors that determine the form of aluminium present in water because the solubility of Al composition varied with pH conditions. The aluminium present in the treated water is largely located in dissolved forms. At alkaline pH, Al(OH)_4_^−^ is the dominant species in aqueous Al [13]. According Frommell et al. [24] pH control and efficient filtration were instrumental for minimizing the residual aluminium concentration in water. Treatment plants coagulation near the pH of minimum aluminium solubility (pH 6.5–7.0) experienced low residual aluminium values. Nowadays, pre-hydrolysed coagulants are used more and more frequently in water treatment plants, among others, polyaluminium chlorides with the general formula Al_n_(OH)_m_Cl_3n-m_. Although the mechanism of coagulation with non-prehydrolysed and pre-hydrolysed aluminium salts is the same, the presence of polymeric forms of aluminium in polyaluminium chloride solutions makes them more stable in water, providing more effective removal of impurities and lower concentrations of aluminium remaining in the treated water. Pre-hydrolysed coagulants are characterised by increased alkalinity (Z) in the range of 15 to 85%, which is determined by the quotient of the mole number OH^-^ to Al^3+^ determined as the alkalinity factor r = [OH^−^]/[Al^3+^] and treated as a measure of the degree of polymerisation. The higher the alkalinity factor (r), the higher the degree of preliminary hydrolysis of the coagulant. The presence of Al_2_(OH)_2_^4+^, Al_3_(OH)_4_^5+^ and Al_13_O_4_(OH)_24_^7+^, aluminium polycations as well as the difference in the structure of precipitated aluminium hydroxide flocs formed during the hydrolysis of pre-hydrolysed coagulants determines their higher efficiency and should ensure lower concentrations of aluminium remaining in the purified water [25,26,27,28,29,30]. Because of the fact that coagulation with aluminium coagulants has undesirable effects, such as aluminium remaining in the purified water that exerts undeniable human health effects, its concentration should be controlled in water treatment plants. The concentration of residual aluminium is influenced by many factors including pH, temperature, and the presence of species such as fluoride, sulphate, silicate, phos-phate, and natural organic matter (NOM). Aluminium solubility has strong temperature dependence, with aluminium concentration increasing as temperature rises. Complexation can enhance aluminium solubility. Fluoride and sulphate form stable complexes with aluminium in acids waters. Natural organic matter (NOM) forms strong bonds with aluminium, often resulting in formation of soluble aluminium-organic species. Because of the complexity and variability of NOM composition, its influence on aluminium solubility is water specific and not well understood [24]. The article presents and discusses the results of studies on the influence of selected properties of aluminium coagulants and especially the effect of Al species in the coagulants on the concentration of aluminium remaining in the purified water and their toxic impact on human health.

## 2. Materials and Methods

### 2.1. Coagulants

Four aluminium coagulants were evaluated in the experiments: aluminium sulphate (VI), sodium aluminate, Flokor 105B and PAX XL10. The coagulants used were commercially available classical hydrolysing coagulants: aluminium sulphate (VI) undergoing water acid hydrolysis and sodium aluminate that undergoes basic hydrolysis as well as commercially pre-hydrolysed polyaluminium chlorides with the trade names: Flokor 105B and PAX XL10 that had different alkalinity and aluminium content. Table 1 shows the characteristics of used coagulants. Aluminium sulphate (VI), sodium aluminate and PAX XL10 are produced by KEMIPOL company in Police (Poland) and Flokor 105B is produced by DEMPOL ECO company in Opole (Poland).

### 2.2. Water Samples Used for Coagulation

The research has been conducted with the use of water collected at the water-treatment plant (WTP) in Zielona Góra. The subject of the study was water being a mixture of groundwater from quaternary formations and surface water. Groundwater after aeration in forced airflow cascades was mixed with surface water at a volume ratio of 1:3 after straining on microsites with a diameter of 10 micrometres. The ranges of values of selected indices of physical-chemical composition of raw water are presented in Table 2. Raw water was characterised by pH in the range from 7.60 to 7.84 and alkalinity from 3.55 to 3.80 mmol/dm^3^. Increased concentrations of organic substances were found in water: TOC varied from 6.781 to 7.282 mgC/dm^3^, DOC from 6.264 to 6.888 mgC/dm^3^ and the mean absorbance value (${\mathrm{UV}}_{254\mathrm{nm}}^{1m}$) determining the content of organic substances characterised by high content of aromatic rings, which are precursors of the by-products of oxidation and disinfection [31] was 15 m^-1^. The colour varied from 6 to 16 mgPt/dm^3^ and the turbidity from 10.6 to 15.0 NTU. The concentration of aluminium in raw water ranged from 0.003 to 0.020 mgAl/dm^3^, including aluminium in the dissolved form to 0.016 mgAl/dm^3^ and in the colloidal form to 0.004 mgAl/dm^3^. The tested water also showed the presence of total iron in the amount from 0.953 to 1.584 mgFe/dm^3^ and iron (II) in the amount from 0.070 to 0.083 mgFe/dm^3^. The calculated mean value of SUVA_254_ (UV_254_/DOC) was 2.34 m^2^/gC, which indicates that there were hydrophilic, hydrophobic, small and macromolecular organic compounds [32,33,34,35]. The value of Zeta potential of colloids present in raw water measured with Zetasizer Nano was −14 mV.

### 2.3. Experimental Procedure of Coagulation

Jar tests were carried out by using a 1 dm^3^ six-place paddle stirrer (Flocculator Kemira 2000, Sweden). Coagulation was carried out in water samples of 1 dm^3^. Fast mixing through 1 min at a speed of 250 rpm. Flocculation through 25 min with an intensity of mixing of 30 rpm. The experiments involved a natural pH from 7.60 to 7.84. The doses of coagulants were expressed in mg Al/dm^3^ and varied from 1 to 5 mg Al/dm^3^. After coagulation the samples were subject to sedimentation process for 1 h. The jar tests were repeated three times and the presented results are the average value.

### 2.4. Analytical Methods

The Al species distribution in the PACls (Flokor 105B, PAXXL10), aluminium sulphate (VI) and sodium aluminate samples were analysed by the Ferron complexation timed spectrophotometry [36,37]. Al(III) reacts with Ferron reagent to form Al-Ferron complex at pH = 5, λ = 370 nm. Agilent Cary 60 spectrophotometer was used to measure the Al-Ferron kinetics. Based on the kinetic difference of reactions between Ferron reagent (8-hydroxy-7-iodo-5-quinoline sulfonic acid) with different hydrolysed species, hydrolysed Al species can be divided into three types: monomeric Al species (Al_a_) (instantaneous reaction: 0–1 min), medium polymerised Al species (Al_b_) (reaction within 120 min) and species of colloidal (Al_c_) (no reaction in 120 min). The physical-chemical composition of all water samples before and after coagulation was determined according to the International Standard methods. The NOM concentration was monitored by measuring the TOC, DOC and UV absorbance at 254 nm. The TOC and DOC were measured using the thermal method and a Shimadzu TOC analyser. DOC was analysed by the TOC Analyser after filtration through 0.45-µm pore diameter membranes. UV absorbance at 254 nm (UV_254_) was measured by a UV-VIS spectrophotometer Agilent Cary 60 using a quartz cell with 1 cm path length after filtration through 0.45-µm membrane. The colour (according to Pt scale), total iron, iron (II) concentrations were determined with the Dr 3900 (HACH Lange) spectrophotometer. Iron (II) was measured using the 1,10 phenanthroline method. Total iron was measured using the same method. As a reducing agent of ferric ions to the ferrous ions, hydroxylamine hydrochloride was used. Aluminium concentrations was determined with the atomic emission spectroscopy (ISP-OES, 5300DV, Perkin Elmer Company, US). Total aluminium and dissolved aluminium concentrations were measured before and after sample filtration through a 0.45-µm membrane, respectively. The temperature and pH of the raw water and the purified water was determined with an WTW Multi Line P4 with an combination pH electrode with temperature corrections. Turbidity was measured using the Hach 2100N Turbidimeter. The alkalinity was determined with a titrimetric method against methyl orange using 0.1 M aqueous solutions of HCl. Measurement of the electrokinetic potential ζ was made in raw water samples after the coagulation process using the Zetasizer Nano Analyser, which calculates the Zeta potential by determining the electrophoretic mobility of the particles using the laser technique of speed measurement based on the Doppler effect.

## 3. Results and Discussion

The total concentration of aluminium remaining in water after the coagulation process is determined by the dissolved products of hydrolysis of aluminium coagulants not used to destabilize colloids, aluminium complexes with organic substances and its hardly soluble connections. The type and concentration of hydrolysis products is influenced mainly by the pH value, temperature and alkalinity of the treated water and the type and dose of coagulant [38].

### 3.1. Coagulant Properties

The percentage of aluminium species in the coagulants, as determined by means of the ferrron method, are shown in Table 3.

The chemical species of hydrolysed Al(III) can be divided into three types according to their reaction kinetics: monomeric Al species (Al_a_) (instantaneous reaction), medium-polymerized Al species (Al_b_) (reaction within 120 min) and species of colloidal (Al_c_) (no reaction in 120 min). Aluminium species distributions were very different. Among the polyaluminium chlorides Al_b_ percentage generally increased with increasing alkalinity of coagulants (Table 1 and Table 3). Flokor 105B possesses higher contents of polymer aluminium forms (Al_b_ = 54%) than PAX XL10 (Al_b_ = 28%). The highest content of colloidal aluminium forms (Al_c_ = 66%) was found in coagulant PAX XL10. Classical hydrolysing coagulants such as aluminium sulphate (VI) and sodium aluminate contained mainly monomeric aluminium species (aluminium sulphate (VI) − Al_a_= 91%; sodium aluminate − Al_a_= 100%). According to literature reports [24] the removal of contaminant by species of Al_a_, Al_b_ and Al_c_ follows the mechanisms of complexation, charge neutralisation and adsorption, respectively.

### 3.2. Influence of Type and Dose of Aluminium Coagulants on the Concentration of Other Aluminium

**Figure 1:**
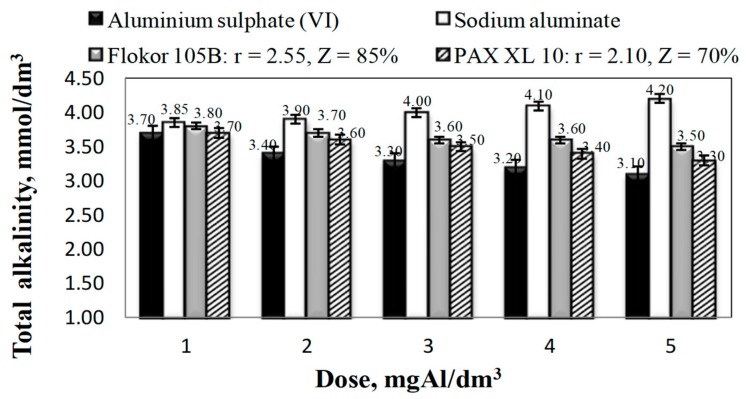
Effect of the type and dose of coagulants on alkalinity in purified water.

**Figure 2:**
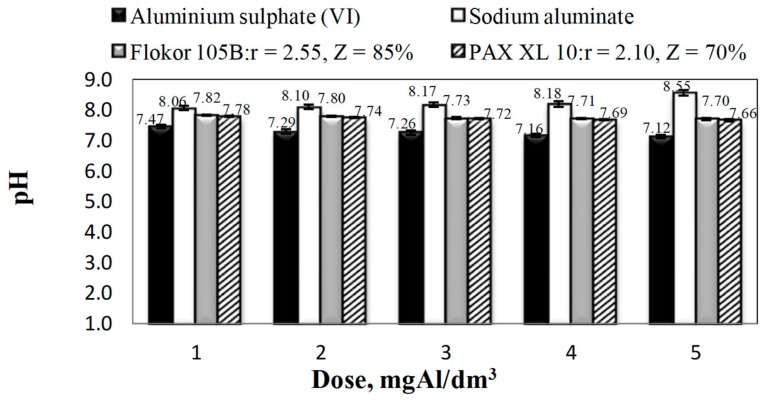
Effect of the type and dose of coagulants on the pH in purified water.

The analysis of the obtained results showed that all the tested coagulants, except for sodium aluminate, decreased the alkalinity and pH of the treated water (Figure 1 and Figure 2).

Sodium aluminate subjected to alkaline hydrolysis caused an increase in alkalinity and pH of the treated water, which increased with the increase in the tested coagulant dose from 1 to 5 mgAl/dm^3^. The alkalinity of treated water after coagulation with sodium aluminate changed from 3.70 to 4.20 mmol/dm^3^ and the pH from 8.06 to 8.55, which was the effect of alkaline hydrolysis of sodium aluminate in water [13]. After coagulation with aluminium sulphate (VI), which undergoes acid hydrolysis [21], the greatest decrease in alkalinity and pH of treated water occurred. The higher the dose of coagulant, the lower the pH in treated water, which decreased from 7.47 to 7.12 and the lower the alkalinity from 3.70 to 3.10 mmol/dm^3^ respectively for the doses from 1 to 5 mgAl/dm^3^.

**Figure 3:**
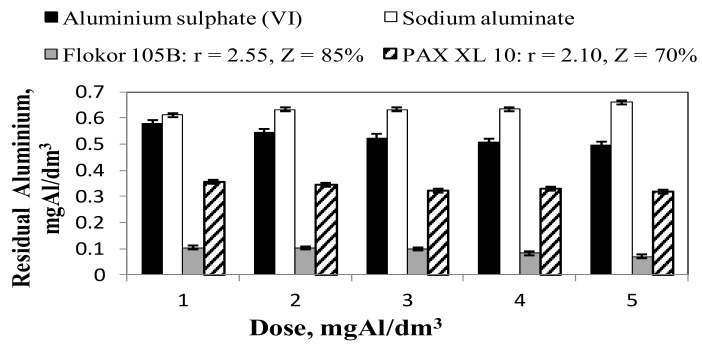
Effect of the type and dose of coagulants on the concentration of aluminium residual in the treated water.

In the case of PAXXL10 and Flokor 105B, i.e., prehydrolysed coagulants, a smaller reduction in alkalinity and pH of water was provided by Flokor 105B coagulant with higher alkalinity and higher alkalinity ratio (Z = 85 %; r = 2.55). Decrease in pH and alkalinity as a result of coagulation with the exception of sodium aluminate increased directly proportionally to the dose of coagulants, whereas in the case of prehydrolysed coagulants it was also inversely proportional to their alkalinity (Figure 1 and Figure 2). The analysis of relations presented in Figure 3 showed that all tested aluminium coagulants caused an increase in aluminium concentration in water after the coagulation process.

**Figure 4:**
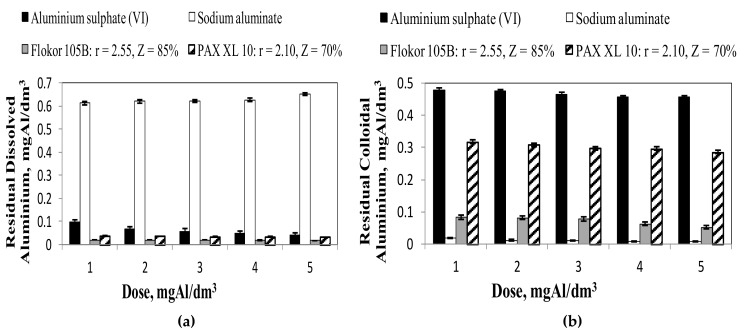
Effect of the type and dose of coagulants on the concentration of residual aluminium in dissolved (**a**) and colloidal (**b**) form in purified water.

The highest concentrations of residual aluminium and dissolved aluminium were found after coagulation with sodium aluminate (Figure 3 and Figure 4a), which undergoes alkaline hydrolysis and increased the pH of treated water in the range from 8.06 to 8.55, creating conditions for the formation of soluble aluminates Al(OH)_4_^−^ [2,19]. The concentration of aluminium remaining after coagulation with sodium aluminate was more than three times higher than the acceptable concentration of aluminium established for water intended for human consumption [8]. The lowest residual aluminium concentrations (Figure 3) meeting the requirements for water intended for human consumption (≤0.2 mgAl/dm^3^) in the whole range of tested doses and very small amounts of residual aluminium in dissolved form (Figure 4a) were found only after coagulation with Flokor 105 B coagulant. Compared to the other tested coagulants, this coagulant had the highest value of the alkalinity factor r = 2.55 (Table 2) and contained the highest amount of polymeric aluminium forms Al_b_ = 54% (Table 3). Therefore, according to literature reports, because of the large amount of polymeric aluminium forms, it formed easily sedimenting chain structure sedimentation sludge [13]. Large floc particles were formed with Flokor 105 B and PAXXL10 whereas the floc particles formed with aluminium sulphate (VI) and sodium aluminate were small at the same dosage (as determined by visual inspection). Matsui and others are of a similar opinion [39] who have shown that the replacement of aluminium sulphate (VI) with polyaluminium chlorides results in the formation of large flocs with good sedimentary properties. The usefulness of the tested aluminium coagulants due to the concentration of residual aluminium and dissolved aluminium, which is easily assimilated by the human body [22], was increased according to the following series: sodium aluminate (Al_a_ = 100%, Al_b_=0) < aluminium sulphate (VI) (Al_a_ = 91%, Al_b_ = 9%) < PAX XL 10 (Al_a_ = 6%, Al_b_=28%, r = 2.10) < Flokor 105B (Al_a_ = 3%, Al_b_ = 54%, r = 2.55). With increasing alkalinity coefficient (r) of pre-hydrolysed coagulants, and thus with increasing share of aluminium polycations (Al_b_) and decreasing share of aluminium monomer forms (Al_a_) in the tested coagulants, the concentration of aluminium remaining in the purified water decreased. According to literature reports [38,39,40] Al_b_ forms play an important role in controlling the residual aluminium concentration therefore charge neutralisation mechanism was found to be the most important factor that determines the residual aluminium concentration directly. Yan et al. [40] reported that the dissolved residual aluminium concentrations for high basicity PACl at acidic and basic pH (<5.5 and >7.5) were lower than that for medium-basicity PACl and AlCl_3_.These investigators attributed the lower aluminium concentrations to the low content of monomeric aluminium species in the high-basicity PACl. The analysis of the obtained results also showed that the highest concentration of aluminium remaining in water after coagulation with sodium aluminate was the result of alkaline hydrolysis and the highest pH in water after coagulation in the range from 8.06 to 8.55, and thus the highest share of Al(OH)_4_^−^ among the products of coagulant hydrolysis. According to Saxena et al. [41] the precipitation of Al(OH)_3_ starts at a pH of about 3, while at a pH of about 8, aluminium hydroxide dissolves into Al(OH)_4_^−^.

**Figure 5:**
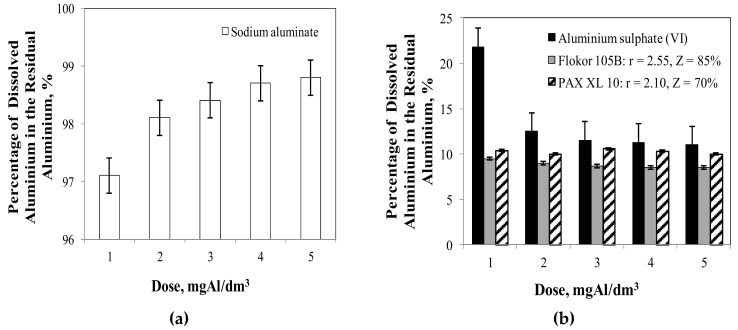
Effect of the dose of aluminium sulphate (VI), PAX XL 10 and Flokor 105B (**a**) and sodium aluminate (**b**) on the content of aluminium dissolved in the total residual aluminium in purified water.

As the pH increases, aluminium hydrolyses according to the follow sequence, Al^3+^→ Al(OH)_2_^+^→ Al(OH)_2_^+^→ Al(OH)_3_→ Al(OH)_4_^−^. A part of aluminium remaining in water after coagulation with the tested coagulants was in colloidal form (Figure 4b). The highest concentrations of aluminium in colloidal form were found in water after coagulation with aluminium sulphate (VI), which undergoes acid hydrolysis and decreased the pH of treated water in the range from 7.47 to 7.12, creating conditions for the formation of Al(OH)_3_. According to literature reports [40] Al_a_, as the main species of aluminium sulphate (VI), has been found to be the most unstable species. Most Al_a_ would hydrolyse immediately after dosing and then react with organic matter in the form of hydroxide. Therefore, sweep flocculation, entrapment and adsorption effect played important roles at neutral condition. The flocs formed by Al_a_ would be easier to be broken and lead to the release of aluminium. According to literature reports [13], aluminium sulphate (VI) forms flocs after entering the water only in a small range of pH from 6.8 to 7.2. If the pH value is lower than 6.8 or higher than 7.2, aluminium sulphate (VI) does not coagulate completely. Therefore, ensuring an optimal pH value for hydrolysing coagulants such as aluminium sulphate (VI) creates the conditions for maximum utilisation of the coagulant used and thus for keeping the minimum quantity in the purified water. This is very important for the health aspect of water consumers, because according to literature reports, aluminium may cause brain changes characteristic for Alzheimer’s disease [1,7]. There are also views on the greater assimilability of aluminium from water than from food [21]. However, opinions on optimal pH ranges for aluminium sulphate (VI) and polyaluminium chlorides are not unanimous. According to Dempsey et al. [42], the optimal pH range for aluminium sulphate (VI) is between 5.5 and 7.0, and for polyaluminium chloride between 4.0 and 8.0. Packham [43], on the other hand, believes that these ranges are appropriately equal: 5.0–6.0 for aluminium sulphate (VI) and 3.0–9.0 for polyaluminium chlorides. The type of aluminium sulphate (VI) hydrolysis products depends to a large extent on the pH. However, the fact of preliminary hydrolysis of polyaluminium chlorides ensures that the aluminium forms produced during the production of these polymer coagulants are introduced into water regardless of the reaction of purified water [38,39,40,41,42,43,44]. The analysis of the obtained results also showed that the highest share of aluminium dissolved in the remaining clay increased with the dose of coagulant, and thus the pH in water after coagulation in the range from 8.06 to 8.55, was found after coagulation with sodium aluminate (Figure 5b)

**Figure 6:**
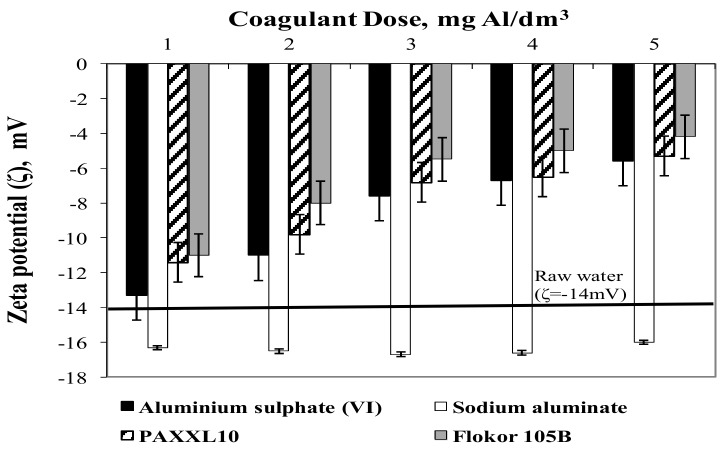
The effect of the type and dose of a coagulant on the change in Zeta potential in the treated.

The lowest share of aluminium dissolved in the remaining aluminium was found after coagulation with pre-hydrolysed poly-aluminium chloride Flokor 105B (Figure 5a), which contained the highest amount of polymeric aluminium forms (Al_b_ = 54%) among the tested coagulants. In case of coagulants pre-hydrolysed PAX XL10 and Flokor 105B and aluminium sulphate (VI) subjected to acid hydrolysis, the proportion of aluminium dissolved in the remaining clay decreased with the dose of coagulants in the range from 1 to 5 mgAl/dm^3^. The analysis of the obtained results also showed that the concentration of aluminium dissolved in purified water increased with the content of monomer forms of aluminium (Al_a_) in the coagulant and was significantly lower after coagulation with polyaluminium chlorides than with non-prehydrolysed coagulants. According to Sieliechi et al. [22], the concentration of dissolved aluminium is associated with the presence of monomer forms of aluminium, therefore the highest concentration of aluminium is recorded during water treatment with hydrolysing aluminium coagulants, while the lowest concentration is recorded when the coagulation process is carried out using reagents with a high content of polymeric forms of aluminium. According Wang et al. [37] for Al_a_, the most reactive species out of the three, it is less efficient and can result in high residual aluminium in the finished water because of the complexation with organic matter. In case of non-prehydrolysed coagulants, the pH value during the coagulation process was decisive for the amount of aluminium remaining in the water (Figure 2 and Figure 4a). Comparing the aluminium concentration in water after coagulation with aluminium sulphate (VI) and sodium aluminate, it can be stated that basically, higher Al_a_ percentage in coagulants leads to bigger residual Al concentration at alkaline conditions (pH > 8). The favourable conditions for hydrolysis of aluminium (pH) allow the formation of the Al_13_ polycation responsible for a good flocculation and reduced residual aluminium including its harmfulness described in several studies [1,7]. The efficiency of the coagulation process of the tested coagulants was also evaluated by measuring the electro-kinetic potential ζ, which determines the stability of the colloidal system (Figure 6).

The highest degree of destabilization of the colloidal system was obtained in water samples after coagulation with Flokor 105B poly-aluminium chloride, which contained the highest amount of polymeric aluminium forms (Al_b_ = 54%). Zeta potential after coagulation with Flokor 105B poly-aluminium chloride varied from −11.0 to −4.20 mV for doses from 1 to 5 mgAl/dm^3^. After coagulation with the least effective sodium aluminate clay coagulant in purified water, an increase in the electro-kinetic potential ζ was observed in the range from −16.0 to −18 mV, while the measured value of the electro-kinetic potential of raw water was −14.0 mV. In the case of organic substances at pH > 8, which was found in water after coagulation with sodium aluminate, the total dissociation of organic substances occurs, thus increasing the negative surface charge.

## 4. Conclusions

Data in the literature suggest that exposure to aluminium via drinking water may be a contributing factor in the development of Alzheimer’s disease and related disorders. The chemical speciation of aluminium in drinking water is very important, as the form of aluminium regulates its solubility, bioavailability and toxicity. Water treatment using aluminium salts, can increase the percentage of dissolved, low molecular weight aluminium species that are chemically reactive and more readily absorbed by human body. The analysis of test results showed that the pre-hydrolysed coagulants provided significantly lower concentrations of aluminium remaining in the treated water than non-prehydrolysed coagulants. Because of the concentration of aluminium remaining in the purified water and the share of aluminium dissolved in the remaining aluminium, which is best absorbed by the human body, the usefulness of the studied aluminium coagulants was increased with an increase in the content of polycationic aluminium forms (Al_b_) and with an decrease in the content of monomeric aluminium forms (Al_a_) in the coagulants tested. The lowest concentrations of residual aluminium meeting the requirements for water intended for human consumption were found only after coagulation with poly-aluminium chloride Flokor 105B containing highest amounts of polymeric forms of aluminium (Al_b_ = 54%). The highest concentrations of remaining aluminium were found in water after coagulation with sodium aluminate which contained the highest amount of monomeric aluminium forms (Al_a_ = 100%) and which also undergoes alkaline hydrolysis creating conditions for the transition of Al(OH)_3_ to soluble Al(OH)_4_^−^ as evidenced by a very high proportion of aluminium dissolved in the remaining aluminium. The highest amounts of aluminium remaining in colloidal form were found in water after coagulation with aluminium sulphate (VI), which undergoes acid hydrolysis and decreased the pH of treated water in the range from 7.47 to 7.12 providing good conditions for the formation of Al(OH)_3_. However, the resulting post-coagulation sediments did not have such good sedimentation properties as in the case of pre-hydrolysed coagulants.

## Figures and Tables

**Table 1:** Selected properties of the coagulants tested (Manufacturer’s specification).

Indicator	Type of Coagulant			
	Aluminium Sulphate (VI)	Sodium Aluminate	PAX XL10	Flokor 105B
Alkalinity ratio, r = [OH^−^]/[Al^3+^]	-	-	2.10	2.55
Alkalinity (Z), %	-	-	70 ± 7	85 ± 5
pH, -	2.4 ± 0.5	12.5 ± 0.5	2.50 ± 0.12	4.20 ± 0.50
Al^3+^, %	4.2 ± 0.2	9.5 ± 0.5	5.0 ± 0.1	7.0 ± 0.5
Fe_tot_, %	<0.007	<0.003	-	-

**Table 2:** Raw water quality characteristics.

Indicator	Unit	Value		
		Minimum	Average	Maximum
pH	-	7.60	-	7.84
Alkalinity	mmol/dm^3^	3.55	3.65	3.80
Turbidity	NTU	10.6	12.8	15.0
Colour	mgPt/dm^3^	6	11	16
TOC	mgC/dm^3^	6.781	6.832	7.282
DOC	mgC/dm^3^	6.264	6.576	6.888
Aluminium total	mgAl/dm^3^	0.003	0.012	0.020
Aluminum colloidal	mgAl/dm^3^	0.000	0.002	0.004
Aluminum dissolved	mgAl/dm^3^	0.003	0.010	0.016
Zeta Potential	mV	−14.00	−14.00	−14.00
Iron total	mgFe/dm^3^	0.953	1.269	1.584
Iron (II)	mgFe/dm^3^	0.070	0.077	0.083
UV_254_	m^−1^	14.25	15.37	16.49
SUVA [UV_254_/DOC]	m^2^/gC	2.18	2.34	2.49

**Table 3:** The degree of polymerization of the aluminium coagulants tested according the conventional ferronometry.

Aluminium Species	Aluminium Sulphate (VI)	Sodium Aluminate	PAX XL10	Flokor 105B
Monomeric Al species (Al_a_), %	91	100	6	3
Polymerized Al species (Al_b_), %	9	0	28	54
Colloidal Al species (Al_c_),%	0	0	66	43
